# Flavonoids, Flavonoid Subclasses and Breast Cancer Risk: A Meta-Analysis of Epidemiologic Studies

**DOI:** 10.1371/journal.pone.0054318

**Published:** 2013-01-18

**Authors:** Chang Hui, Xie Qi, Zhang Qianyong, Peng Xiaoli, Zhu Jundong, Mi Mantian

**Affiliations:** 1 Research Center for Nutrition and Food Safety, Third Military Medical University, Chongqing Key Laboratory of Nutrition and Food Safety, Chongqing, China; 2 Department of Public Health, School of Preclinical Medicine, Chengdu Medical College, Chengdu, Sichuan, China; University of Washington, United States of America

## Abstract

**Background:**

Studies have suggested the chemopreventive effects of flavonoids on carcinogenesis. Yet numbers of epidemiologic studies assessing dietary flavonoids and breast cancer risk have yielded inconsistent results. The association between flavonoids, flavonoid subclasses (flavonols, flavan-3-ols, etc.) and the risk of breast cancer lacks systematic analysis.

**Objective:**

We aimed to examine the association between flavonoids, each flavonoid subclass (except isoflavones) and the risk of breast cancer by conducting a meta-analysis.

**Design:**

We searched for all relevant studies with a prospective cohort or case-control study design published before July 1^st^, 2012, using Cochrane library, MEDLINE, EMBASE and PUBMED. Summary relative risks (RR) were calculated using fixed- or random-effects models. All analyses were performed using STATA version 10.0.

**Results:**

Twelve studies were included, involving 9 513 cases and 181 906 controls, six of which were prospective cohort studies, and six were case-control studies. We calculated the summary RRs of breast cancer risk for the highest vs lowest categories of each flavonoid subclass respectively. The risk of breast cancer significantly decreased in women with high intake of flavonols (RR = 0.88, 95% CI 0.80–0.98) and flavones (RR = 0.83, 95% CI: 0.76–0.91) compared with that in those with low intake of flavonols and flavones. However, no significant association of flavan-3-ols (RR = 0.93, 95% CI: 0.84–1.02), flavanones (summary RR = 0.95, 95% CI: 0.88–1.03), anthocyanins (summary RR = 0.97, 95% CI: 0.87–1.08) or total flavonoids (summary RR = 0.98, 95% CI: 0.86–1.12) intake with breast cancer risk was observed. Furthermore, summary RRs of 3 case-control studies stratified by menopausal status suggested flavonols, flavones or flavan-3-ols intake is associated with a significant reduced risk of breast cancer in post-menopausal while not in pre-menopausal women.

**Conclusions:**

The present study suggests the intake of flavonols and flavones, but not other flavonoid subclasses or total flavonoids, is associated with a decreased risk of breast cancer, especially among post-menopausal women.

## Introduction

Breast cancer is the leading cause of cancer death among women in Europe and North America. Almost 1.4 million women were diagnosed with breast cancer worldwide in 2008 and approximately 459,000 deaths were recorded [1], [2]. More than 2.5 million breast cancer survivors live in United States currently, and the number is expected to grow to 3.4 million by 2015 [3]. The National Cancer Institute (NCI) has recognized that prevention is a critical component in minimizing the number of individuals afflicted with cancer [4]. Recent reports suggest that approximately one-third of the most common cancers in western countries can be prevented by eating a healthy, plant-based diet; being physically active; and maintaining a healthy weight [5]. Epidemiologic studies and systematic analysis suggest diets rich in fruits and vegetables are associated with a reduced risk of cancer, in particular cancers of epithelial origin such as those of the mouth, colon, rectum [6], lung [7], and breast [8], [9]. As consumption of fruits and vegetables has been associated with a reduced risk of human cancers especially breast cancer [10], [11], dietary flavonoids, a group of more than 5 000 different polyphenolic compounds, have been identified as potential cancer-preventive components of fruits and vegetables [12], [13].

**Figure 1:**
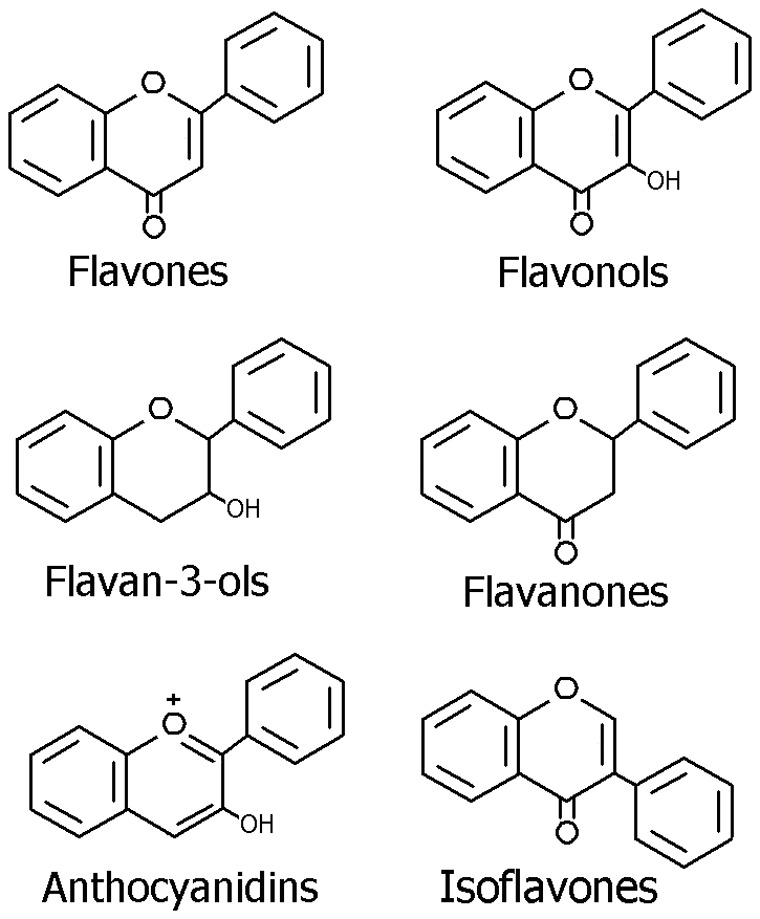
Structures of six flavonoid subclasses.

Dietary flavonoids occur ubiquitously in plant foods, and can be categorized into six major subclasses based on their range and structural complexity: flavonols, flavones, flavan-3-ols, flavanones, anthocyanins and isoflavones (Figure 1) [14]–[16]. As reviewed before by Hooper L *et al* (Table 1), in western diet, major flavonoids included in these subclasses are quercetin, myricetin and kaempferol for flavonols, hesperitin and narigerin for flavanones, epicatechin and catechin for flavan-3-ols, apigenin and luteolin for flavones, cyanidin, delphinidin and malvidin for anthocyanidines, and genistein and daizeina for isoflavones [16]–[18]. Flavonols mainly exist in onions, broccoli, tea, and various common fruits, flavones in aromatic herbs, celery, and chamomile tea, flavan-3-ols in cocoa, red wine, grapes, apples, green tea, and other fruits, flavanones in oranges and other citrus fruits, anthocyanidines in colored berries, black currants, and isoflavones in soy food [19]–[21].

**Table 1:** Flavonoid subclasses, food sources and intakes [14].

Flavonoid subclasses	Example compounds	Major dietary sources	Estimated daily intakes
Flavonols	Quercetin, kaempferol, myricetin, and isorhamnetin	Onions, broccoli, tea, and various fruits	mg/d12.9
Flavones	Luteolin, apigenin, and tangeretin	Herbs (especially parsley), celery, and chamomile tea	1.6
Flavanones	Naringenin, hesperetin	Citrus fruit including oranges and grape fruit	14.4
Flavan-3-ols	Catechin, epicatechin, epigallocatechin	Cocoa or dark chocolate, apples, grape, red wine, and green tea	156.9
Anthocyanidins	Cyanidin, delphinidin, pelargonidin, andmalvidin	Colored berries and other fruit, especially cranberries, black currants, and blueberries	3.1
Isoflavones	Genistein, daidzein, and glycitein	Soy products including fermented products, eg, tofu, tempeh, miso, and soy protein isolate	1.2 (US and Netherlands) 25–50 (Asia)

In decades, studies have suggested the chemopreventive effects of flavonoids on carcinogenesis, the anticancer activity of dietary flavonoids has become an important and interesting topic. Yet in fact, a number of epidemiologic studies assessing the association between dietary flavonoid intake and the risk of breast cancer have yielded inconsistent results and have identified controversial evidence [22]–[34]. One possible explanation is that different flavonoid subclasses in diet may have different properties and effects *in vivo*. So it is important to elucidate the different role of each flavonoid subclass in the chemopreventive effect of these dietary compounds. However no systematic analysis has been performed to assess the association between dietary intake of flavonoid subclasses and the risk of breast cancer. Thus, we aimed to examine the association of each flavonoid subclass intake with the risk of breast cancer by performing a meta-analysis of epidemiologic studies. Given that many reviews have been conducted to assess the association between the dietary isoflavone intake and the breast cancer risk, we excluded this issue in the present study.

## Methods

### Search Strategy

We conducted a systematic search of literature published before July 1^st^ 2012 using the Cochrane Library, MEDLINE, and EMBASE Databases and the following search terms: “flavonoid”, “flavonols”, “flavones”, “flavanones”, “flavan-3-ols”, “flavanols”, “anthocyanidins”, “phytoestrogens”, “polyphenolic compounds” and “breast cancer”. We also performed a manual search using reference lists of original articles and relevant reviews. Only full-length original journal articles were considered and no attempt was made to include abstracts or unpublished studies.

### Study Selection

Studies were eligible for our analysis if: (1) the study design is cohort or case-control study; (2) data related to dietary consumption or exposure assessment (blood/urinary levels) of total flavonoids or one of flavonoid subclasses (isoflavones excluded) were available; (3) the association of flavonoids or one of flavonoid subclasses with breast cancer risk was specifically evaluated; (4) relatively complete assessment of total flavonoids or flavonoid subclass intake was performed; (5) relative risk (RR), hazard ratio (HR), or odds ratio (OR), and corresponding 95% confidence intervals (95% CI) were available. Because isoflavones have been studied extensively, including meta-analysises, studies focusing on isoflavones alone were not included in the present study. Originally, we included RCTs in our search criteria, but because there were no RCTs on flavonoids, no RCTs are included in the present study.

### Data Extraction

We recorded study characteristics as follows: (1) name of the first author and publication year; (2) country or origin; (3) study design (cohort or case-control study); (4) mean length of follow-up; (5) number of cases and controls; (6) assessment of exposure, especially the database for assessment of flavonoid intake; (7) exposures to flavonoids; (8) media of flavonoids intakes; (9) RR, HR or OR from the most fully adjusted model for the highest versus the lowest flavonoids exposure and their 95% CI; (10) confounders adjusted for in multivariate analysis.

### Statistical Analysis

We investigated the associations between intakes of each flavonoid subclass and the risk of breast cancer separately. Homogeneity of effect size across studies was tested by Q statistics (P<0.10). We also computed the I^2^, a quantitative measure of inconsistency across studies. If substantial heterogeneity exists, the random-effects model is appropriate; otherwise, the fixed-effects model is preferred [35]. A sensitivity analysis was conducted using both fixed- and random-effects models to evaluate the robustness of results. The potential publication bias was examined by the funnel plot and Egger’s test [36] (P<0.10). All analyses were performed using STATA version 10.0 (Stata Corp, College Station, Texas, USA). A P value <0.05 was considered statistically significant, except where otherwise specified.

## Results

### Characteristics of the Included Studies

The twelve studies [22]–[34] met the inclusion criteria after our complete review. Characteristics of these studies are presented in Table 2. The studies included in the final analysis had 9 513 cases and 181 906 controls.

**Table 2:** Characteristics of the included studies.

Author, year and region	Study design	Mean follow-up	Cases/controls	Assessment of exposure	Flavonoids exposure and media of intake	OR or RR(95% CI)			Adjustments
		(year)			(mg/d)	Total	Premenopausal	Postmenopausal	
Wang L 2009, U.S.A	Cohort	1995–2007	1351 (38408)	SFFQ, Databases published in US and Europe	Total flavonoids(19.13)	1.03(0.85 1.25)			age, race, energy intake, menopausal status, hormone replacement therapy, intake of fruit and vegetables et al.
Arts ICW 2002,U.S.A	Cohort	1986–1998	1069 (34651)	SFFQ, Database from Netherlands	Flavan-3-ols(14.8)	1.04(0.84 1.28)			age, education level, race, multivitamin use, menopausal status, BMI, energy intake, smoking habit, physical activity.
Adebamowo CA 2005, U.S.A	Cohort	1991–1999	710 (90630)	FFQ, Databasepublished inEurope	Flavonols(17.1)	1.05(0.83 1.34)			age, parity, age at first pregnancy, age at menarche, menopausal status, BMI, energy intake, alcohol consumption, height, smoking, et al.
Knekt P 2002, Finland	Cohort	1967–1994	125 (4647)	QFIQ, Databases published in Finland	Total flavonoids(24.2)	1.23(0.72 2.10)			age, geographic area, occupation, smoking, BMI
Goldbohm 1998, Netherlands	Cohort	1986–1991	605(2 203)	SFFQ, Database from Netherlands	Total flavonoids(29.1)	1.02(0.72 1.44)			age, education level, race, multivitamin use, menopausal status, BMI, energy intake, smoking habit.
Knekt P 1997, Finland	Cohort	1967–1991	87 (4699)	QFIQ, Databasepublished inNetherland	Total flavonoids(nd)	0.72(0.36 1.48)			sex, age, geographic area, occupation, BMI, energy intake, smoking, vit C and E, cholesterol, β-carotene, fiber, SFA, MUFA,PUFA
Luo JF 2010, Shanghai China	Nested case-control	1997–2004	352/701	Urinary excretion analysis	Flavonols(nd) Flavan-3-ols(nd)	1.04(0.73 1.48)1.12(0.77 1.63)			age, education, age at menarche, age at 1st live birth, months of breastfeeding, smoking, et al.
Dai Q 2002, Shanghai China	Population- based case-control	1996–1998	250/250	Urinary excretion analysis	Flavanones(nd)	1.04(0.66 1.63)	1.53(0.77 3.04)	0.79(0.41 1.51)	age at first live birth, ever diagnosed with fibroadenoma, total meat intake, and physical activity level.
Luisa TS 2008, Mexico	Hospital- based case-control	1994–1996	141/141	SFFQ, Databases published in Mexico	Flavonols(27.8) Flavones(2.5) Flavan-3-ols(7.9)	0.48(0.21 1.08) 0.60(0.27 1.37) 0.80(0.38 1.70)	0.49(0.19 1.23) 0.49(0.19 1.29) 1.22(0.48 3.08)	0.21(0.07 0.60) 0.29(0.10 0.82) 0.63(0.25 1.62)	age, energy intake, lifetime lactation
Fink BN 2007, New York	Population- based case-control	1996–1997	1434/1440	FFQ, Database from USDA	Total flavonoids Flavonols(9.8) Flavones(0.13) Flavan-3-ols(162) Flavanones(31.2) Anthocyanidins(3.15)	0.88(0.69 1.12) 0.75(0.59 0.95) 0.73(0.57 0.93) 0.85(0.67 1.08) 0.89(0.70 1.12) 0.91(0.72 1.15)	1.12(0.72 1.74) 1.38(0.88 2.15) 1.07(0.70 1.65) 1.21(0.78 1.86) 0.80(0.53 1.21) 1.08(0.71 1.63)	0.75(0.56 1.01) 0.54(0.40 0.73) 0.61(0.45 0.83) 0.74(0.55 0.99) 1.00(0.75 1.34) 0.85(0.64 1.14)	age,energy intake.
Bosetti C 2005, Italy	Hospital- based case-control	1991–1994	2569/2588	FFQ, Database from USDA	Flavonols(18.6) Flavones(0.5) Flavan-3-ols(36.4) Flavanones(33.7) Anthocyanidins(10.4)	0.80(0.66 0.98) 0.81(0.66 0.98) 0.86(0.71 1.05) 0.95(0.79 1.15) 1.09(0.87 1.36)	0.90(0.80 1.02) 0.87(0.76 0.99) 0.94(0.85 1.05) 0.98(0.85 1.13) 1.14(1.00 1.31)	0.97(0.89 1.05) 0.90(0.81 1.00) 0.92(0.84 1.00) 0.93(0.82 1.05) 1.04(0.93 1.17)	age,study center, education, parity, alcohol consumption, nonalcohol energr intake.
Peterson J 2003, Athens, Greece	Hospital- based case-control	1989–1991	820/1548	SFFQ, Database from USDA	Flavonols(19.4) Flavones(0.4) Flavan-3-ols(23.5) Flavanones(33.5) Anthocyanidins(20.9)	0.91(0.78 1.06) 0.87(0.77 0.97) 0.93(0.78 1.11) 0.96(0.87 1.07) 0.94(0.81 1.09)			age, place of birth, parity, age at first pregnancy, age at menarche, menopausal status, BMI, energy intake, alcohol consumption.

BMI: body mass index; 95% CI: 95% confidence intervals; FFQ: food frequency questionnaire; nd: no detection; QFIQ: quantitative food intake questionnaire; SFFQ: semiquantitative food frequency questionnaire; USDA: U.S.Department of Agriculture; SFA: saturated fatty acids, MUFA: monounsaturated fatty acids, PUFA: polyunsaturated fatty acids.

The selected studies were published between 1997 and 2010 spanning 13 years, and all of them were published in English. Among these 12 studies, 6 were prospective cohort studies, 1 was nested case-control study, 2 were population-based case-control studies, and 3 were hospital-based case-control studies; moreover, 4 studies were from USA, 2 from Finland, 2 from China, and the rest were respectively from Netherlands, Mexico, Italy and Greece. The exposure assessments of flavonoids in 10 studies were made by food frequency questionnaire or by quantitative food intake questionaire, and in 2 studies were measured by urinary excretion analysis. Most individual studies were adjusted for a wide range of potential confounders, including age, race, education, energy intake, BMI, physical activity, parity, smoking, alcohol, and hormone replacement therapy.

### Flavonoid Subclasses and Breast Cancer Risk

**Figure 2:**
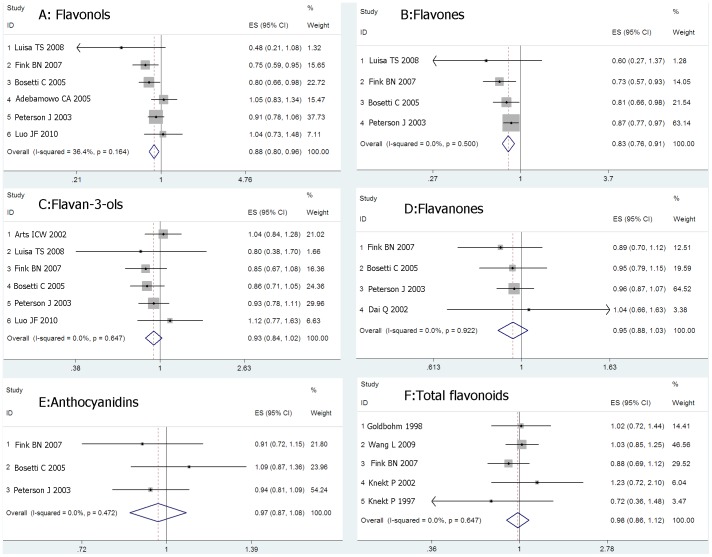
Meta-analysis of studies examining association between flavonoids consumption and risk of breast cancer.

We identified 6 studies of flavonols intake and breast cancer risk, 4 studies of flavones, 6 studies of flavan-3-ols, 4 studies of flavanones, 3 studies of anthocyanins, and 5 studies of total flavonoids. We calculated the summary RR using fixed- or random-effects models respectively. As shown in Figure 2, no substantial heterogeneity existed across studies of the flavonoid subclasses. Overall, the risk of breast cancer significantly decreased in women with highest intakes of flavonols (summary RR = 0.88, 95% CI: 0.80–0.98) and flavones (summary RR = 0.83, 95% CI: 0.76–0.91) by 12% and 17% respectively, compared with that in those with lowest intakes of flavonols and flavones. However, no significant association of flavan-3-ols (summary RR = 0.93, 95% CI: 0.84–1.02), flavanones (summary RR = 0.95, 95% CI: 0.88–1.03), anthocyanins (summary RR = 0.97, 95% CI: 0.87–1.08) or total flavonoids (summary RR = 0.98, 95% CI: 0.86–1.12) with breast cancer risk was observed.

### Effect of Menopausal Status on Association between Flavonoid and Breast Cancer

Summary RRs of 4 case-control studies were stratified by menopausal status [20], [22], [23]. As shown in Table 3, significant associations of flavonols, flavones and flavan-3-ols intakes with reduced risk of breast cancer were observed in post-menopausal while not in pre-menopausal women. Menopausal status may contribute to the association between flavonoids and breast cancer risk. However, there were significant heterogeneities among studies of flavonols and flavones in post-menopausal women, and of flavan-3-ols in pre-menopausal women. Furthermore, no significant association between flavanones intake and breast cancer risk was observed in either post-menopausal or pre-menopausal women.

**Table 3:** Results of stratified analyses by menopausal status.

Menopause status	Summary RR(95% CI)	P for heterogeneity	I^2^, %
**Flavonols**			
Pre-menopause	0.92 (0.82 1.03)	0.081	60.3
Post-menopause	0.92 (0.85 0.99)	0.000	90.4
**Flavones**			
Pre-menopause	0.88 (0.77 1.00)	0.323	11.5
Post-menopause	0.86 (0.77 0.94)	0.008	79.3
**Flavan-3-ols**			
Pre-menopause	0.96 (0.86 1.06)	0.000	0.474
Post-menopause	0.90 (0.83 0.98)	0.286	20.2
**Flavanones**			
Pre-menopause	0.98 (0.86 1.11)	0.281	21.3
Post-menopause	0.94 (0.84 1.05)	0.791	0.0

### Publication Bias

**Figure 3:**
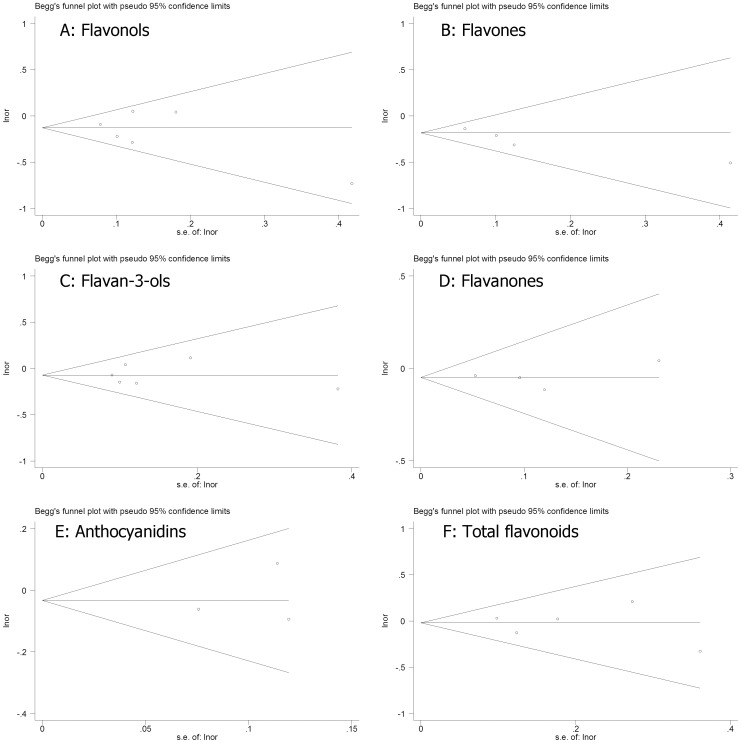
Funnel plot of flavonoids consumption and risk of breast cancer.

As shown in Figure 3, results from Egger’s tests indicated little evidence of publication bias in these studies (flavonols: P = 0.571, flavones: P = 0.106, flavan-3-ols: P = 0.890, flavanones: P = 0.964, anthocyanins: P = 0.449, and total flavonoids: P = 0.853).

## Discussion

Studies have suggested that plant flavonoids have many biological benefits, such as the antioxidant, anti-inflammatory, anti-tumor [37] and anti-atherosclerosis effects [38], [39]. Cancer preventive phytochemicals, especially flavonoids, have been shown to suppress or block cancer progression by a variety of mechanisms [40], [41]. More attention is given to preventing colon, rectum, lung, prostate or breast cancer through daily diet because of the chemoprotective effects of dietary flavonoids and other phytochemicals. However, most of the cancer preventive effects of phytochemicals, including flavonoids, were shown in animal and cell culture studies; human clinical trials examining the chemopreventive potential of phytochemicals are lacking. In fact, some epidemiologic studies assessing the association between the flavonoid intake and the breast cancer risk have yielded inconsistent results. Moreover, different dietary flavonoid subclasses, which vary in chemical structures and bioactivities, may have different chemopreventive effects on breast cancer. The present meta-analysis of population studies supports a significant association of flavonols and flavones intake with a reduced risk of breast cancer. However, neither the total flavonoids nor the other flavonoid subclasses intake has been found to be associated with the breast cancer risk. More studies are warranted to confirm the results. The findings likely provide useful insight and evidence that can be used by registered dietitians and other healthcare professionals when discussing diet and cancer prevention with patients.

In establishing flavonoids as one of the contributors to the protective effects, the very first step is to estimate flavonoid intake from various dietary sources [21]. Yet dietary flavonoids are composed of a great variety of polyphenolic compounds which widely exist in plant foods, so it is difficult to assess the intake of total flavonoids and flavonoid subclasses. Part of the inconsistencies of epidemiological studies may be attributable to the difficulty in measuring intake levels of flavonoids. The estimated daily intake of total flavonoids in the same country may differ in different studies, suggesting that some heterogeneity may exist in dietary assessment of flavonoids intake. Estimation of flavonoid intake from dietary sources has been feasible since 2003 when the U.S. Department of Agriculture (USDA) released the database for the flavonoid content of selected foods. Since then, many articles have been published in which flavonoid intake in various subpopulation groups was estimated from relatively large, current databases of flavonoid concentration data. Furthermore, biomarkers such as urinary excretion or plasma metabolite levels could complement dietary assessment of the bioavailability of these dietary compounds. However, information is still limited on the intake of flavonoids and each flavonoid subclass in the United States and worldwide. More carefully designed studies should be performed to improve the method and database for assessing dietary flavonoids intake.

Menopausal status and estrogen-receptor (ER) status, as effect modifiers, may greatly effect the association between the flavonoid intake and breast cancer risk. Some studies showed that the association between the intake of soy isoflavone and the reduced risk of breast cancer incidence or recurrence was stronger in post-menopausal women than in premenopausal women [42], [43]. Although the other flavonoid subclasses have weaker phytoestrogen activity than isoflavones, the menopausal status and ER status also influence their association with breast cancer. The present analysis indicates a significant association of flavonol, flavone and flavan-3-ol intake with the reduced risk of breast cancer in post-menopausal but not in pre-menopausal women. The possible mechanism might partially lie in that flavonoids affect the ovarian synthesis of sex hormones or the alteration of other menstrual cycle characteristics [44], [45]. Although flaonoids, especially isoflavones, are most widely recognized for their weak estrogenic activity, they have a variety of other biologic activities that may influence cancer risk, such as antioxidant, antiproliferative, [46] and antiangiogenic activities [47] as well as inhibiting the effects of cytokines, growth factors, and several enzymes [48], [49]. The anticancer effects of flavonoids may be exerted by the combination of a variety of biologic activities, and would be influenced by some established risk factors for cancer such as alcohol consumption [50], smoking status, energy intake, menopausal status, use of hormonal treatment for menopause et al [51], [52]. Therefore, the chemoprevention of flavonoids may be varied among different subpopulation. More carefully designed studies should be performed to investigate the association of phytochemicals with cancer.

### Conclusions

The present study suggests the intakes of flavonols and flavones, but not the other flavonoid subclasses or total flavonoids, can potentially contribute to breast cancer prevention, especially among post-menopausal women. More studies are needed to confirm the findings.
